# Effect of arbuscular mycorrhizal fungi and phosphorus on drought-induced oxidative stress and 14-3-3 proteins gene expression of *Populus cathayana*

**DOI:** 10.3389/fmicb.2022.934964

**Published:** 2022-08-11

**Authors:** Yanyan Han, Wenrui Zhang, Tingying Xu, Ming Tang

**Affiliations:** ^1^College of Forestry, Northwest A&F University, Xianyang, China; ^2^Boone Pickens School of Geology, Oklahoma State University, Stillwater, OK, United States; ^3^State Key Laboratory for Conservation and Utilization of Subtropical Agro-Bioresources, Guangdong Laboratory for Lingnan Modern Agriculture, College of Forestry and Landscape Architecture, South China Agricultural University, Guangzhou, China

**Keywords:** arbuscular mycorrhizal fungi, phosphorus metabolism, drought stress, reactive oxygen species homeostasis, 14-3-3 proteins

## Abstract

The application of arbuscular mycorrhizal fungi (AM fungi) and phosphorus (P) can improve plant growth under drought stress by upregulating the antioxidant system and osmotic accumulation. The 14-3-3 protein can respond to different abiotic stresses such as low P and drought. The purpose of this experiment was to study the effects of AM fungi (*Rhizophagus intraradices*) inoculation on reactive oxygen species (ROS) homeostasis, P metabolism, and 14-3-3 gene expression of *Populus cathayana* at different P levels and drought stress (WW: well-watered and WD: water deficit). Under WD conditions, AM fungi inoculation significantly increased the P content in leaves and roots, but the benefit in roots is limited by the level of P addition, and the roots may have more alkaline phosphatase and phytase under P stress, and these activities in the rhizosphere soil inoculated with AM fungi were stronger. Under WD conditions, the activities of catalase (leaf and root) and peroxidase (root) inoculated with AM fungi were significantly higher than those without inoculation and decreased with P addition. 14-3-3 genes, *PcGRF10* and *PcGRF11*, have a positive correlation with the antioxidant system, osmotic regulation, and P metabolism, which may be more significant after inoculation with AM fungi. Our results provide new insights into the mechanism of ROS homeostasis and P metabolism in mycorrhizal plants under drought stress.

## Introduction

Global climate change has led to harsh growing environments, making plants face more abiotic stress and reduces tree yields (Meena et al., 2017). Drought is a very severe and common environmental factor limiting tree yield (Mathur et al., 2019). Plants can produce reactive oxygen species (ROS), including hydroxyl ions (OH^–^), hydrogen peroxide (H_2_O_2_), and superoxide radical (O_2_^–^) with toxic effects that affect physiological metabolism under drought stress (Mittler, 2002). The various physiological and molecular processes play an important role in plant stress tolerance, including antioxidant enzymes such as superoxide dismutase (SOD), catalase (CAT), and peroxidase (POD) (Hanin et al., 2016). As the carrier of nutrients, water plays a vital role in the migration and absorption of nutrients. Water deficiency affects the nutrient uptake of plants and nutrient deficiency inhibits water utilization and ultimately reduces plant growth (Rubin et al., 2017).

Previous studies have shown that phosphorus (P) deficiency exacerbates drought stress (Sardans and Penuelas, 2012). P is the second most important macronutrient in plant growth and development after nitrogen (N), and the bioavailable amount of P is more affected by drought stress than N (Mariotte et al., 2020). Phosphate deficiency and drought stress co-occur in some arid and semi-arid regions of the world. Drought stress is predicted to increase worldwide, which could change the nutrient redistribution in soils and can have a significant impact on forest trees by reducing the supply and absorption of nutrients (Sanaullah et al., 2012). P availability decreases with the decrease of soil moisture content (Hira and Singh, 1977). P application can alleviate the adverse effects of drought by promoting root growth, nutrient uptake, and water use efficiency under the condition of water shortage (Waraich et al., 2011).

Many studies have demonstrated that beneficial soil microorganisms, including arbuscular mycorrhizal (AM) fungi, in the plant rhizosphere can improve drought resistance by establishing a symbiotic relationship with plants (Coleman-Derr and Tringe, 2014). AM fungi inoculation alleviates stress damage to plant growth and biomass accumulation by reducing the production of toxic free radicals and upregulating the activity of antioxidant enzymes (Huang et al., 2014; Chitarra et al., 2016). AM fungi play an important role in the P utilization of plants. In exchange for carbon uptake from plants (Bücking and Shachar-Hill, 2005), AM fungi provide mineral nutrients to the hosts, especially P with low mobility in soils (Kiers et al., 2011). The hyphae of AM fungi can absorb P from the inaccessible soil pore space to plant roots and transfer it to host plants (Zheng et al., 2015). AM fungi can also improve the availability of soil P by hydrolyzing inorganic P and mineralizing organic P (Hetrick, 1989). Studies have shown that mycorrhizal symbiosis can promote P uptake in plants, and in turn, good mycorrhizal symbiosis is closely related with the P concentration in the environment (Smith and Smith, 2011). The application of an appropriate concentration of P can enhance the root colonization of AM fungi and promote the growth and development of plants (Mathimaran et al., 2007). The results of Begum et al. (2020) showed that the application of AM fungi and P significantly ameliorated the growth decline of tobacco under drought stress by upregulating the antioxidant system, osmotic accumulation, and root activity.

The 14-3-3 proteins are a class of regulatory proteins with highly conserved sequences and are widely found in various eukaryotes (Sun et al., 2018). As a intersection of the signal network in plant response to abiotic stress, these proteins can respond to a variety of different stresses (Oecking and Jaspert, 2009). Recent findings highlight the role of 14-3-3 proteins in ABA signaling and plant drought tolerance (Sun et al., 2013; Xu et al., 2018). However, the exact function of the 14-3-3 protein in response to nutrient deficiency is unclear. At present, there are some studies on the role of tomato 14-3-3 gene family members under low P stress, mainly manifested in the aspects of starch accumulation and activation of plasma membrane H^+^-ATPase (Xu et al., 2012a,b; Zhang et al., 2018a).

*Populus cathayana* Rehd. is a rapidly growing dioecious plant, and males are better than females under environmental stress such as drought and nutrient deficiency (Chen et al., 2014; Han et al., 2018). Previous studies have demonstrated that males of *P. cathayana* adopt energy-saving strategies to cope with P deficiency by metabolic and physiological analysis, resulting in stronger tolerance to P deficiency than females (Zhang et al., 2019). The enhancement effect of P on the drought resistance of males was more effective than females under severe drought stress (Xia et al., 2020b). Based on this, we designed an experiment to determine the effect of different P concentrations on the drought resistance of *P. cathayana* males inoculated with AM fungi, focusing on AM fungi colonization, dry matter accumulation, P metabolism, oxidative damage, antioxidant enzyme activity, and 14-3-3 gene expression. We hypothesized that the *P. cathayana* inoculated AM fungi at different P levels and drought stress would affect (1) the P metabolism; (2) the reactive oxygen species homeostasis; and (3) the expression of 14-3-3 family genes.

## Materials and methods

### Experiment materials

*Rhizophagus intraradices* (BGC AH01) was provided by the Beijing Academy of Agriculture and Forestry Sciences, China. Maize (*Zea mays* L.) was propagated in the laboratory of the Forestry College of Northwest A&F University to obtain a mixture of spores (12 per gram of inoculum), mycelium, and root segments as the test inoculum. The sand through a 2-mm sieve was washed and sterilized for 3 h at 170°C in the oven. The soil samples were collected at Northwest A&F University and were autoclaved at 121°C for 2 h, air-dried, and then mixed with sand in equal volume as the cultivation substrate.

The experimental plants were male cuttings (length: 10 cm, diameter: 1–1.3 cm) of annual *P. cathayana* Rehd., purchased from Datong Linbo Nursery Stock Professional Cooperative in Qinghai Province. The seedling raising process is as follows (Supplementary Figure 1A): after surface disinfection with 0.05% potassium permanganate for 3 h, the cuttings were soaked in sterile water for 3 days and the water was changed every 12 h. The cuttings germinated and developed leaves for 20 days in the seedling tray. Then the buds with consistent growth were broken off and inserted into the seedling pot (1 plant/pot, 30 g cultivation substrate/pot). Phosphorus-free Hoagland nutrient solution was poured once a week during the period. After the seedlings were grown for a month, the plastic film outside the seedling pot was cut off and they were moved into a flowerpot (upper diameter: 12.5 cm, bottom diameter: 8 cm, height: 10 cm) that was filled with 950 g cultivation substrate. Meanwhile, half of the seedlings was inoculated with 20 g AM fungi inoculum and the other half was inoculated with 20 g inactivated AM fungi inoculum. The cultivation substrate of each flowerpot reached 1 kg.

### Experimental design and treatments

The experiment adopted a three-factor completely randomized block design (Supplementary Figure 1B): AM fungi (AM: AM fungi inoculation, NM: non-inoculation), P (P0: 0 mg P, P50: 50 mg P, P100: 100 mg P, and P150: 150 mg P) (Xia et al., 2020a), and drought (WW: well-watered, WD: water deficit), with a total of 16 treatments, 3 replicates for each treatment, 2 pots for each replicate, yielding a total of 96 pots plants. Seedlings were treated with P immediately after inoculation. KH_2_PO_4_ solution (54 mmol/L) was poured for 3 days (P0: 0 mL, P50: 10 mL, P100: 20 mL, and P150: 30 mL per day, and the remaining volume was filled with distilled water), then the P content reached 0, 50, 100, and 150 mg, respectively. To maintain K^+^ at a constant concentration, KH_2_PO_4_ was replaced by KCl. an appropriate amount of KCl was added to meet the 150 mg P (KH_2_PO_4_) amount of K for each treatment. During this period, the daily watering amount reached 70–75% of the maximum field water capacity, and 20 mL phosphorus-free Hoagland nutrient solution was watered every 10 days. After 40 days of inoculation and P treatment, seedlings were treated with drought stress: one-half of each treatment was kept in a well-watered condition (70–75% maximum field capacity) and the other half was treated with water deficit (30–35% maximum field capacity). Water was controlled by weighing at 17:00 once a day before drought stress treatment, and water was controlled twice a day at 8:00 and 17:00 during drought stress treatment. No phosphorus was applied during drought treatment. The potted seedlings were cultivated in a plastic greenhouse, the average temperature was about 19°C at night and 30°C during daylight (in the hot summer, from May to August), the humidity was controlled at 30–40%, and the light was natural light in summer. Plant samples and soil samples were harvested for further assays after 2 months of drought treatment, and plant samples and soil samples from 2 pots of each replicate were mixed. Plant samples were stored in two parts: the frozen leaves and roots were grounded into powder with liquid nitrogen for enzyme-related analysis and the dried leaves and roots were grounded into powder for element determination. The soil samples were air-dried and passed through a 1-mm sieve.

### Mycorrhizal colonization

After harvesting, the roots of each treatment were washed with running water to remove the cultivation substrate. The fibrous roots were cut off with clean scissors and then stored in a test tube filled with FAA fixative (formalin/glacial acetic acid/70% alcohol = 1:1:18). The fibrous roots were removed before staining, rinsed with tap water, and stained in 0.05% (w/v) trypan blue (Phillips and Hayman, 1970). Stained root segments were kept overnight and checked under the light microscope (Olympus BX43F, Tokyo, Japan). The extent of colonization was confirmed using the grid method, with 200 intersections observed per treatment (Giovannetti and Mosse, 1980).

### Growth measurements

Before harvesting, 3 plants were randomly selected from each treatment for plant height determination. Then the roots, stems, and leaves were weighed and collected in an envelope bag. A total of 1 g root, stem, and leaf samples were taken as fresh weight and dried to constant weight in an oven at 70°C, respectively. Biomass of the above-ground and underground parts of the plant was calculated in proportion. The root activity was analyzed by the triphenyl tetrazolium chloride (TTC) method (Wang et al., 2006), expressed as TTC reduction intensity.

### P content

The dried sample (leaf: 0.05 g, root: 0.05 g, soil: 0.1 g) and 7 mL concentrated nitric acid (superior pure) were added into a digestive tube and then transferred to a microwave digestion apparatus (Multiwave PRO, Austria) for digestion. After complete digestion, the acid in the digestive tube remained about 1 mL by evaporating at a high temperature (160°C). The sample digests were diluted with deionized water (18 mΩ × cm) to reach a volume of 50 mL. P content was determined with a continuous flow chemistry analyzer (Systea company, Flowsys, FR, Italy). Soil available P (soil: 0.5 g) was extracted with 0.5 mol/L NaHCO_3_ and determined by the molybdenum–antimony–scandium colorimetric method (Bray and Kurtz, 1945).

### Phosphatase activity

Phosphatase activity was determined by the 4-nitrophenyl phosphate disodium salt (PNPP) method (Tabatabai and Bremner, 1969; Feng et al., 2002). The frozen leaves and roots and the air-dried soil samples (leaf: 0.2 g, root: 0.2 g, and soil: 0.5 g) were extracted with 0.2 mol/L glacial acetic acid-sodium acetate buffer (pH 5.0) and 0.2 mol/L borax-sodium hydroxide buffer (pH 10.0) for the determination of acid phosphatase (ACP) and alkaline phosphatase (ALP), respectively. A control set with no substrate was needed to measure soil phosphatase activity. Finally, colorimetry was performed at 410 nm using a full-wavelength microplate reader (Thermo Fisher Scientific, Multiskan GO, Helsinki, Finland).

### Phytase activity and phytic acid content

The frozen leaves and roots (0.1 g) and the air-dried soil samples (1.0 g) were used for phytase and phytic acid measurements. The phytase of leaves and roots was extracted according to the method of Chen et al. (2008), then the activity was determined by the increase of inorganic P using the method of Wyss et al. (1999). Finally, colorimetry was performed at 700 nm using a full-wavelength microplate reader. The soil phytase activity was carried out according to the method of Zhang et al. (2013) and the absorbance was measured at 415 nm. The content of phytic acid was determined according to the method of Chen et al. (2008), and the colorimetry was carried out at 500 nm.

### Pigment content

A total of 0.05 g of fresh leaves were extracted with 95% ethanol to obtain chlorophyll and then the absorbance was measured with a microplate reader at 665, 649, and 470 nm wavelengths according to the method from Gao (2006). 0.05 g of fresh leaves were extracted with a methanol solution containing 1% HCL (v/v), and the absorbance was measured at the wavelengths of 530 and 652 nm. The concentrations of chlorophyll a (Ca), chlorophyll b (Cb), carotenoid (Cx.c), and anthocyanin (Ant) were calculated according to the following formula, and their contents were calculated according to the sample volume and quality.


Ca=13.95A-6656.88A649

Cb=24.96A-6497.32A665

Cx.c=(1000A-4702.05Ca-114.8Cb)/245

Ant=A-5300.25A652

### Reactive oxygen species-scavenging enzymes and oxidative stress markers

A total of 0.05 g frozen leaves or roots and 1.6 mL pre-cooled 50 mmol/L phosphate-buffered saline (PBS) (pH 7.8) that included 1% polyvinylpyrrolidone were added to the 2 mL Eppendorf tube. The samples were then centrifuged at 12,000 *g* for 20 min at 4°C and the supernatant was used as the test solution. The activities of SOD and POD were measured according to Gao’s (2006) method. The activity of CAT was determined according to the method of Aebi (1984), but the CAT reaction solution was changed to 100 mL PBS (0.15 M, pH 7.0) and 0.03 mL 30% H_2_O_2_. The rate of O_2_^–^ production was determined according to the method of Ke et al. (2007). The H_2_O_2_ content was detected by potassium iodide spectrophotometry (Chakrabarty and Datta, 2008). The malondialdehyde (MDA) content was measured using the thiobarbituric acid method described by Kramer et al. (1991) at wavelengths of 450, 532, and 600 nm by a full-wavelength microplate reader. The proline content was determined by the acid ninhydrin method according to the description of Gao (2006).

### RNA extraction and RT-qPCR analysis

The total RNA of leaves and roots was extracted using the E.Z.N.A.^®^ Plant RNA Kit (Omega Bio-Tek, Norcross, GA, United States). The synthesis of first-strand cDNA was carried out with 1 μg RNA by using the reverse transcription kit (HiScript^®^ II Reverse Transcriptase, Vazyme). Then the cDNA was diluted 5 times for quantitative real-time PCR (qRT-PCR) analysis. *PcGLL* and *Pctublin* were the internal reference genes of *P. cathayana* qRT-PCR. The primers for qRT-PCR are shown in Supplementary Table 1. The reaction system was 10 μL: 5 μL 2 × ChamQ SYBR qPCR Master Mix (Vazyme), 1 μL forward primer, 1 μL reverse primer, 1.5 μL DEPC water, and 1.5 μL cDNA. The reaction conditions were: 95°C for 3 min; 39 cycles at 95°C for 10 s, 58°C for 20 s, and 72°C for 20 s; followed by 95°C for 10 s, and then 61 cycles of 5 s increasing from 65°C to 95°C by an increment of 0.5°C for each cycle. The 2^–ΔΔ*Ct*^ method (Livak and Schmittgen, 2001) was used to calculate the relative quantitative values of different genes.

### Statistical analysis

IBM SPSS Statistics 26.0 (SPSS Inc., Chicago, IL, United States) was used to analyze the experimental data. Independent-sample Duncan test and three-way analysis of variance (ANOVA) (factors: AM fungi inoculation, drought stress, and P addition) (*P* < 0.05) were used to compare the differences among treatments. All data were reported as means ± standard deviation (SD). The principal component analysis (PCA), orthogonal partial least-squares discrimination analysis (OPLS-DA), correlation heatmap analysis, and redundancy analysis (RDA) were carried out in the Wekemo Bioinformatics Cloud^1^.

## Results

### Mycorrhizal colonization

The total mycorrhizal colonization rate of *P. cathayana* had no significant difference in response to P concentration under WW and WD conditions (Table 1). The arbuscular colonization rate under the WD condition was significantly higher than that of WW. The arbuscular formation at P50 was significantly higher than the other P levels in the WW treatment, and at P100 it was the highest, but there was no significant difference from the other three P levels in the WD treatment. The colonization rate of hyphae and vesicles increased with the increase of P concentration under WW conditions, but no significant difference was observed under WD conditions. The colonization rates of arbuscular, vesicle, and hyphae were significantly correlated with drought.

**TABLE 1:** Mycorrhizal colonization rates of *P. cathayana* under different P addition and water treatment.

Treatment		AM fungi structural colonization (%)			
		Arbuscular	Vesicle	Hypha	Total
AM + WW	P0	29.63 ± 2.12c	78.55 ± 6.78ab	45.99 ± 6.59c	87.81 ± 3.35a
	P50	40.12 ± 1.49b	78.09 ± 6.75ab	67.44 ± 7.38b	88.89 ± 4.24a
	P100	25.77 ± 3.08c	84.10 ± 14.83a	71.30 ± 3.96b	89.97 ± 7.03a
	P150	28.86 ± 2.79c	85.65 ± 8.83a	89.81 ± 1.60a	95.83 ± 1.60a
AM + WD	P0	72.53 ± 4.30a	73.46 ± 7.64ab	93.21 ± 0.71a	94.60 ± 1.34a
	P50	75.62 ± 6.30a	62.50 ± 8.42b	87.50 ± 8.03a	89.51 ± 6.96a
	P100	78.70 ± 8.57a	64.04 ± 11.58b	93.83 ± 6.70a	93.52 ± 4.63a
	P150	70.83 ± 6.68a	61.88 ± 6.96b	86.73 ± 3.01a	88.58 ± 2.09a
Drought		***	**	***	NS
P		NS	NS	***	NS

AM + WW represents well-watered and AM fungi inoculation treatment, and AM + WD represents water deficit and AM fungi inoculation treatment. P0, P50, P100, and P150 represent different P content of 0, 50, 100, and 150 mg, respectively. The same lowercase letters following the means indicate non-significant differences in corresponding treatments by Duncan’s multiple range test (P < 0.05). Significance mark of three-factor ANVOA: **P < 0.01, ***P < 0.001, NS is not significant; the name of three-factor ANVOA: AM represents inoculation with R. intraradices; drought represents drought treatment; P represents different P addition treatments. The value is expressed as the mean ± standard deviation, n = 3.

### Growth and root activity

**FIGURE 1:**
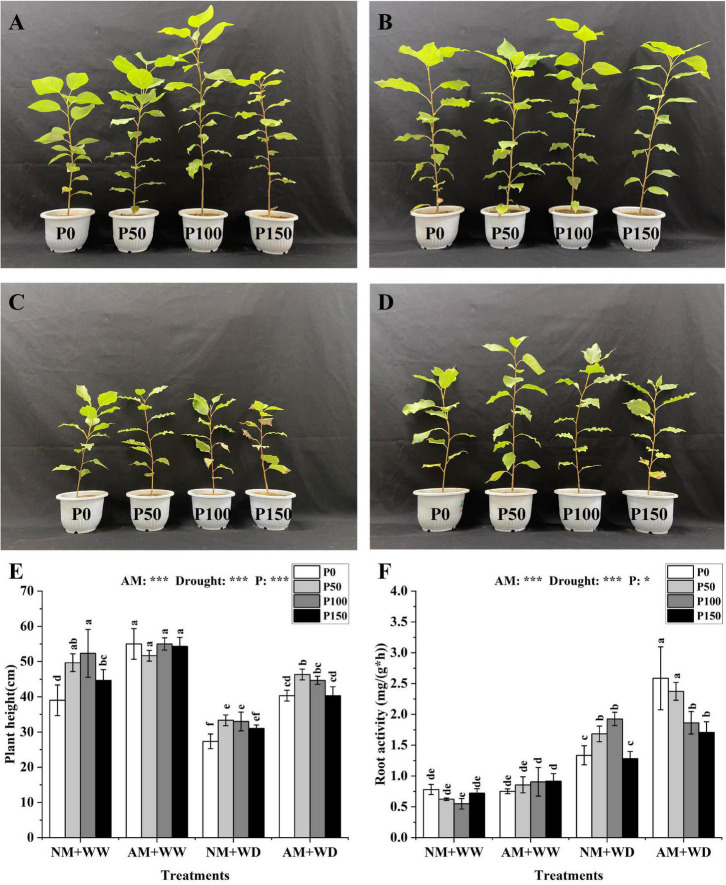
Growth graph, plant height, and root vitality of *Populus cathayana* seedlings inoculated with AM fungi under different P addition and water treatment. **(A)** The growth graphs of NM + WW; **(B)** the growth graphs of AM + WW; **(C)** the growth graphs of NM + WD; **(D)** the growth graphs of AM + WD; **(E)** plant height; and **(F)** root vitality. NM + WW, well-watered and non-inoculation treatment; AM + WW, well-watered and AM fungi inoculation treatment; NM + WD, water deficit and non-inoculation treatment; AM + WD, water deficit and AM fungi inoculation treatment. P0, P50, P100, and P150 represent different P content of 0, 50, 100, and 150 mg, respectively. The same lowercase letters following the means indicate non-significant differences in corresponding treatments by Duncan’s multiple range test (*P* < 0.05). Significance mark of three-factor ANVOA: *P < 0.05, ***P < 0.001, the name of three-factor ANVOA: AM represents inoculation with *R. intraradices*; drought represents drought treatment; P represents different P addition treatments. The value is expressed as the mean ± standard deviation, *n* = 3.

The growth states of *P. cathayana* under the “NM + WW”, “AM + WW”, “NM + WD” and “AM + WD” treatments were shown in Figures 1A–D. The plant height of *P. cathayana* that was inoculated with AM fungi was significantly higher than those of non-mycorrhizal seedlings at all P levels under the WD condition, and it increased by 47.56, 39.00, 35.35, and 30.11% at P0, P50, P100, and P150 levels, respectively. However, AM inoculation only significantly increased plant height at P0 and P150 levels under WW conditions. The plant height was very significantly correlated with AM fungi, drought, and P concentration (*P* < 0.001) (Figure 1E). The root activity of *P. cathayana* mycorrhizal seedlings had no significant difference in the WW treatment. However, the root activity of AM group compared with the NM group increased by 93.52, 40.93, and 33.00% at P0, P50, and P150 levels and decreased by 3.29% at P100 level under the WD condition, respectively. The root activity was significantly correlated with P concentration (*P* < 0.05) and had a very significant correlation with AM fungi and drought (*P* < 0.001) (Figure 1F).

### Biomass

The biomass (shoot, root, and total) of the mycorrhizal *P. cathayana* seedlings compared to non-mycorrhizal seedlings was increased under the WW condition, but there were no significant differences under the WD conditions at the same P level (Supplementary Figures 2A–C). In the WW treatment, the AM group increased by 39.89, 12.75, 26.94, and 16.28% in shoot biomass; 98.46, 43.70, 6.01, and 28.75% in root biomass; and 58.83, 24.22, 18.42, and 20.93% in total biomass at P0, P50, P100, and P150 levels than the NM group, respectively. The root-to-shoot biomass ratio of the mycorrhizal seedlings at the same P level compared with non-mycorrhizal seedlings increased (without P100) under the WW condition but significantly decreased under the WD condition (Supplementary Figure 2D). The dry weight of *P. cathayana* seedlings was significantly correlated with AM fungi, drought, and P to varying degrees.

### P content

**FIGURE 2:**
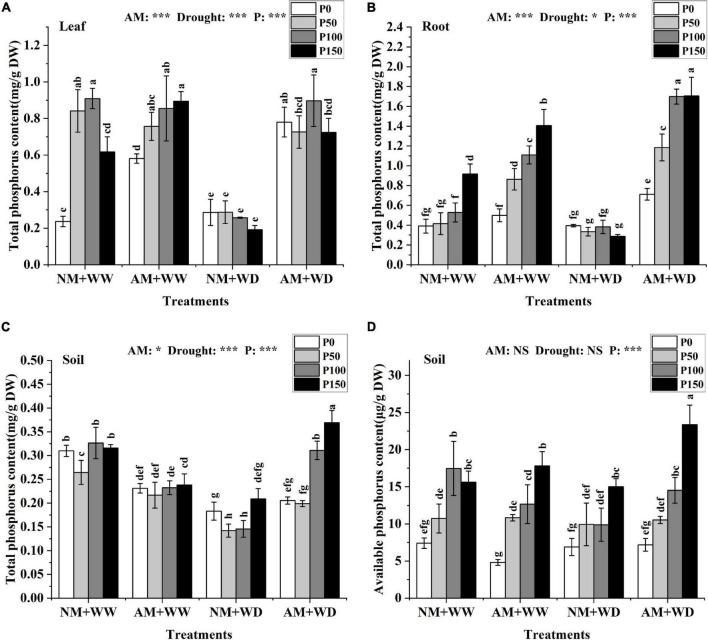
P content of *P. cathayana* seedlings and soil inoculated with AM fungi under different P addition and water treatment. **(A)** Leaf total P content; **(B)** root total P content; **(C)** soil total P content; **(D)** soil available P content. NM + WW, well-watered and non-inoculation treatment; AM + WW, well-watered and AM fungi inoculation treatment; NM + WD, water deficit and non-inoculation treatment; AM + WD, water deficit and AM fungi inoculation treatment. P0, P50, P100, and P150 represent different P content of 0, 50, 100, and 150 mg, respectively. The same lowercase letters following the means indicate non-significant differences in corresponding treatments by Duncan’s multiple range test (*P* < 0.05). Significance mark of three-factor ANVOA: *P < 0.05, ***P < 0.001, NS is not significant; the name of three-factor ANVOA: AM represents inoculation with *R. intraradices*; drought represents drought treatment; P represents different P addition treatments. The value is expressed as the mean ± standard deviation, *n* = 3.

Under the WW condition, the total P content in the leaves (without P150 in the NM group) and roots increased with the increase of P level regardless of inoculation or not. In the “NM + WD” treatment, the total P content had no significant difference in leaves and roots at different P levels. However, AM fungi inoculation significantly improved the total P content in leaves, roots, and rhizosphere soil of *P. cathayana* seedlings at different P levels under the WD conditions. The total P content of the mycorrhizal seedlings compared with that without inoculation, respectively, increased by 172.56, 152.45, 248.10, and 276.76% in leaves; 80.13, 254.25, 343.69, and 492.39% in roots; and 12.18, 39.97, 113.45, and 76.56% in rhizosphere soil at P0, P50, P100, and P150 levels under the WD conditions (Figures 2A–C). The total P content in the leaves, roots, and rhizosphere soil of *P. cathayana* was significantly correlated with AM fungi, drought, and P. The soil available P content increased with the increase of P levels regardless of water conditions and AM fungi inoculation or not, and it was significantly correlated with P (*P* < 0.001) (Figure 2D).

### Phosphatases

**FIGURE 3:**
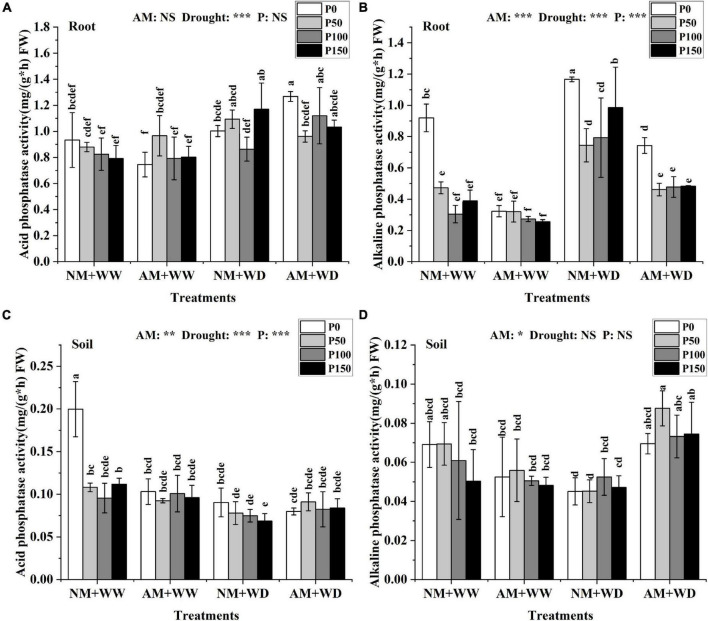
Phosphatase content of *P. cathayana* roots and soil inoculated with AM fungi under different P addition and water treatment. **(A)** Acid phosphatase activity in the root; **(B)** alkaline phosphatase activity in the root; **(C)** acid phosphatase activity in the soil; **(D)** alkaline phosphatase activity in the soil. NM + WW, well-watered and non-inoculation treatment; AM + WW, well-watered and AM fungi inoculation treatment; NM + WD, water deficit and non-inoculation treatment; AM + WD, water deficit and AM fungi inoculation treatment. P0, P50, P100, and P150 represent different P content of 0, 50, 100, and 150 mg, respectively. The same lowercase letters following the means indicate non-significant differences in corresponding treatments by Duncan’s multiple range test (*P* < 0.05). Significance mark of three-factor ANVOA: *P < 0.05, **P < 0.01, ***P < 0.001, NS is not significant; the name of three-factor ANVOA: AM represents inoculation with *R. intraradices*; drought represents drought treatment; P represents different P addition treatments. The value is expressed as the mean ± standard deviation, *n* = 3.

AM fungi, drought, and P had no significant effect on the ACP activity in leaves of *P. cathayana* seedlings (Supplementary Figure 3A). In the WW treatment, the ACP activity of roots in the AM group and NM group had no significant differences at the same P level. Under the WD condition, AM fungi inoculation significantly promoted the ACP activity of roots at P0 and P100 levels, which increased by 26.45 and 29.70% compared with that without inoculation, respectively (Figure 3A). The ACP activity of the rhizosphere soil under “NM + WW + P0” conditions was significantly higher than that in other treatments (Figure 3C).

The ALP activity of leaves was decreased with the increase of P concentration level regardless of inoculation and drought conditions, except for the “NM + WW + P0” treatment (Supplementary Figure 3B). The ALP activity in roots at the same P level was higher in the WD group than the WW group and lower in AM group than the NM group. In the WD treatment, the ALP activity of roots inoculated with AM fungi compared with the non-inoculation decreased by 36.27, 37.98, 39.78, and 51.00% at the P0, P50, P100, and P150 levels, respectively (Figure 3B). The ALP activity of the rhizosphere soil in the AM group at the same P level compared to the NM group decreased under WW conditions but increased under WD conditions, increasing by 54.10, 93.81, 39.43, and 57.84% at P0, P50, P100, and P150 levels, respectively (Figure 3D).

### Phytase and phytic acid

**FIGURE 4:**
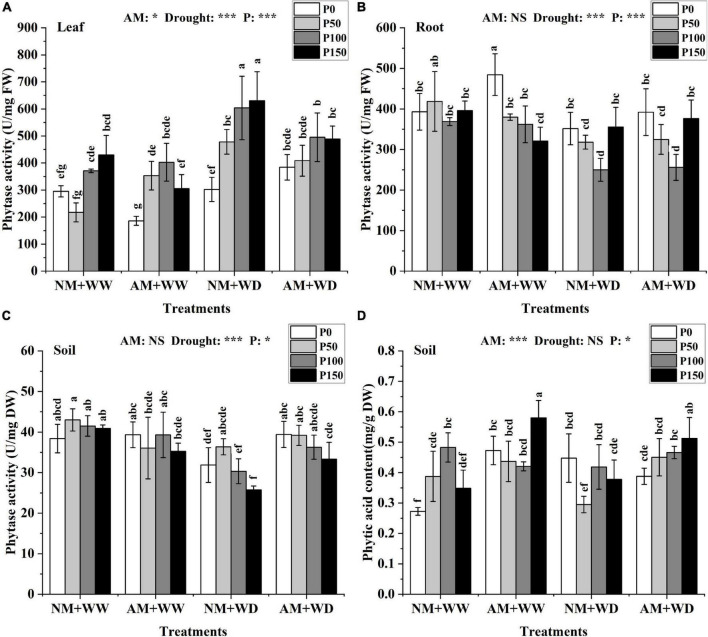
Phytase activity of *P. cathayana* seedlings and phytase activity and phytic acid content of soil inoculated with AM fungi under different P addition and water treatment. **(A)** Phytase activity in leaf; **(B)** phytase activity in root; **(C)** phytase activity in soil; **(D)** phytic acid content in the soil. NM + WW, well-watered and non-inoculation treatment; AM + WW, well-watered and AM fungi inoculation treatment; NM + WD, water deficit and non-inoculation treatment; AM + WD, water deficit and AM fungi inoculation treatment. P0, P50, P100, and P150 represent different P content of 0, 50, 100, and 150 mg, respectively. The same lowercase letters following the means indicate non-significant differences in corresponding treatments by Duncan’s multiple range test (*P* < 0.05). Significance mark of three-factor ANVOA: *P < 0.05, ***P < 0.001, NS is not significant; the name of three-factor ANVOA: AM represents inoculation with *R. intraradices*; drought represents drought treatment; P represents different P addition treatments. The value is expressed as the mean ± standard deviation, *n* = 3.

In general, phytase activity increased in leaves and decreased in roots and rhizosphere soil with the addition of P level in the treatment of “NM + WW,” “AM + WW,” “NM + WD,” and “AM + WD” (Figures 4A–C). The phytase activity of the roots in the NM group and AM group showed the same trend in different P levels and no significant difference in the same P concentration under the WD condition (Figure 4B). The phytase activity of the inoculated soil compared with the non-inoculated soil increased by 23.72, 7.79, 19.61, and 29.48% at P0, P50, P100, and P150 levels under the WD condition, respectively (Figure 4C).

The phytic acid content of leaves and roots at the same P level had no significant differences between the AM and NM groups under the WW and WD condition (Supplementary Figures 3C,D). The phytic acid content of the inoculated soil compared with the non-inoculated soil increased by 73.49, 12.67, and 66.45% at P0, P50, and P150 levels under the WW condition, and increased by 52.57, 11.29, and 35.68% at P50, P100, and P150 levels under the WD condition (Figure 4D).

### Pigment distribution

The chlorophyll contents (a and b) of the leaves of *P. cathayana* seedlings in the “NM + WW” and “AM + WW” treatment decreased with the increase of P addition level, and that of the “NM + WW + P0” treatment was significantly higher than other treatments. However, AM and NM groups had no significant differences at the same P level under the WW and WD conditions (Supplementary Figures 4A,B). Carotenoid and anthocyanin content of the leaves inoculated with AM fungi at the same P level compared with the no-inoculation had no significant difference under the WW condition. The carotenoid content of the AM group and NM group had a similar trend with the increase in P addition level and no significant difference at the same P level under the WD condition. The anthocyanin content of the leaves in the AM group significantly decreased by 28.69 and 31.51% at P100 and P150 levels compared to the NM group under WD conditions (Supplementary Figures 4C,D).

### Reactive oxygen species-scavenging enzymes

**FIGURE 5:**
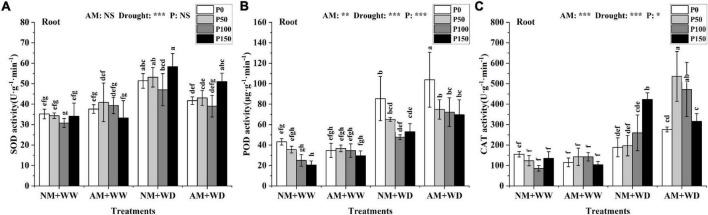
Antioxidant enzyme activity in the root of *P. cathayana* seedlings inoculated with AM fungi under different P addition and water treatment. **(A)** SOD activity; **(B)** POD activity; **(C)** CAT activity. NM + WW, well-watered and non-inoculation treatment; AM + WW, well-watered and AM fungi inoculation treatment; NM + WD, water deficit and non-inoculation treatment; AM + WD, water deficit and AM fungi inoculation treatment. P0, P50, P100, and P150 represent different P content of 0, 50, 100, and 150 mg, respectively. The same lowercase letters following the means indicate non-significant differences in corresponding treatments by Duncan’s multiple range test (*P* < 0.05). Significance mark of three-factor ANVOA: *P < 0.05, **P < 0.01, ***P < 0.001, NS is not significant; the name of three-factor ANVOA: AM represents inoculation with *R. intraradices*; drought represents drought treatment; P represents different P addition treatments. The value is expressed as the mean ± standard deviation, *n* = 3.

The leaves’ SOD activity at the same P level had no significant difference between the AM group and NM group in the WW and WD treatment, and the same results were found in the roots of the WW treatment (Supplementary Figure 5A and Figure 5A). The SOD activity of the “NM + WD” treatment was significantly higher than the “NM + WW” treatment in the roots. The “NM + WD” and “AM + WD” treatments had the same trend with the increase of the P addition level, and the AM group decreased by 18.82, 19.05, 17.12, and 12.48% than the NM group at P0, P50, P100, and P150 levels, respectively (Figure 5A).

The POD activity in leaves and roots at the same P level had no significant difference between the “NM + WW” and “AM + WW” treatment (Supplementary Figure 5B and Figure 5B). Compared with the NM group, the POD activity in leaves of the AM group at P0, P50, P100, and P150 levels decreased by 29.67, 15.68, 18.47, and 12.44% under the WD condition, respectively. The POD activity of the roots in the WD group was significantly higher than in the WW group and decreased with the increase in P concentration. However, the POD activity of the roots, respectively, increased by 21.75, 15.04, 51.38, and 31.24% in AM group than the NM group at P0, P50, P100, and P150 levels under the WD condition.

The CAT activity of the leaves had no significant difference among the “NM + WW,” “AM + WW,” and “NM + WD.” But that for the “AM + WD” was significantly higher than the first three, showing a decreasing trend with the increase in P concentration. The CAT activity of the “AM + WD” compared with “NM + WD” increased by 266.47, 204.29, 124.58, and 74.08% at P0, P50, P100, and P150 levels, respectively (Supplementary Figure 5C). The CAT activity of roots increased significantly under the WD condition than the WW and increased with the increase of P concentration in the NM group. The CAT activity of the AM group increased by 46.14, 172.15, and 81.55% at P0, P50, and P100 levels and decreased by 25.39% at the P150 level under the WD condition (Figure 5C).

### Oxidative stress markers

**FIGURE 6:**
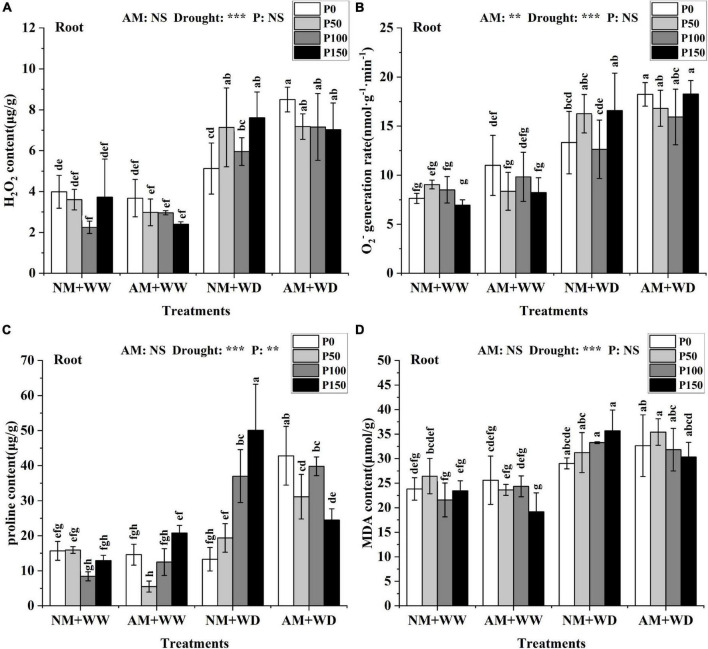
Oxidative damage material in the root of *P. cathayana* seedlings inoculated with AM fungi under different P addition and water treatment. **(A)** H_2_O_2_ content; **(B)** O_2_^–^ generation rate; **(C)** proline content; **(D)** MDA content. NM + WW, well-watered and non-inoculation treatment; AM + WW, well-watered and AM fungi inoculation treatment; NM + WD, water deficit and non-inoculation treatment; AM + WD, water deficit and AM fungi inoculation treatment. P0, P50, P100, and P150 represent different P content of 0, 50, 100, and 150 mg, respectively. The same lowercase letters following the means indicate non-significant differences in corresponding treatments by Duncan’s multiple range test (*P* < 0.05). Significance mark of three-factor ANVOA: **P < 0.01, ***P < 0.001, NS is not significant; the name of three-factor ANVOA: AM represents inoculation with *R. intraradices*; drought represents drought treatment; P represents different P addition treatments. The value is expressed as the mean ± standard deviation, *n* = 3.

The H_2_O_2_ content in leaves and roots of *P. cathayana* under WD conditions was significantly higher than that under WW conditions. But the H_2_O_2_ content in leaves and roots at the same P level had no significant difference between the AM group and NM group under the WW and WD condition, except for P100 in leaves and P0 in roots under WD conditions (Supplementary Figure 6A and Figure 6A). The H_2_O_2_ content of the AM group compared with the NM group significantly increased by 37.31% at the P100 level in the leaves and by 65.79% at the P0 level in roots under the WD condition. There was no significant difference in the generation rate of O_2_^–^ of the leaves at the same P level in the four treatment groups of “NM + WW,” “AM + WW,” “NM + WD,” and “AM + WD,” but the generation rate of O_2_^–^ of the P0 level in each group was higher than that in the other three P levels (Supplementary Figure 6B). The generation rate of O_2_^–^ of the roots under the WD condition was significantly higher than that under the WW condition. The generation rate of O_2_^–^ in the roots of the AM group compared with the NM group was significantly increased by 36.89% at the P0 level under the WD condition (Figure 6B).

There was no significant difference in the proline content of leaves under the WW condition, but it was significantly higher in the WD group than in the WW group. The proline content in leaves of the AM group than in the NM group increased by 53.04, 502.85, and 26.52% at the P0, P100, and P150 levels and decreased by 35.07% at the P50 level in the WD treatment (Supplementary Figure 6C). The proline content in the roots of the AM group than in the NM group increased by 222.35, 60.80, and 7.59% at the P0, P50, and P100 levels and decreased by 51.11% at the P150 level under the WD condition (Figure 6C). The MDA content in leaves and roots at the same P level had no significant difference between the AM group and the NM group under the WW and WD condition except for P0 in leaves under WW conditions (Supplementary Figure 6D and Figure 6D). The content of MDA of the roots in the WD group was significantly higher than in the WW group.

### Relative expression of 14-3-3 family genes

The expression of some 14-3-3 genes in the leaves and roots of *P. cathayana* seedlings was affected by drought stress, AM fungi inoculation, and P addition. The expression levels of *PcGRF10*, *PcGRF11*, and *PcGRF12* in leaves and *PcGRF10* and *PcGRF11* in roots were significantly higher than the other genes (Supplementary Figures 7, 8). Under the WW condition, the relative expression levels of *PcGRF10* in the AM group at P0, P50, P100, and P150 levels increased by 114.96, 115.24, 485.50, and 434.20% in the leaves and 0.95, −11.89, 24.46, and 121.42% in the roots than in the NM group; and the *PcGRF11* increased by 131.77, 123.52, 493.40, and 432.97% in the leaves and 70.98, 46.94, 80.81, and 223.39% in the roots. Under the WD condition, the relative expression levels of *PcGRF10* in the AM group compared with the NM group increased by 112.91, 35.87, 84.03, and 81.34% in leaves and −0.95, 23.40, −22.87, and −31.55% in the roots at P0, P50, P100, and P150 levels, respectively; and the *PcGRF11* in the AM group at P0, P50, P100, and P150 levels increased by 145.14, 39.79, 247.24, and 96.85% in leaves and 25.36, 28.93, 7.98, and 13.41% in roots than in the NM group. The relative expression levels of *PcGRF10* and *PcGRF11*, respectively, reached 13.82–22.53 and 17.13–25.78 in leaves and 2.57–3.25 and 5.99–7.43 in roots in the treatment of “AM + WD.”

### Principal component analysis and orthogonal partial least-squares discrimination analysis of physiological indicators

**FIGURE 7:**
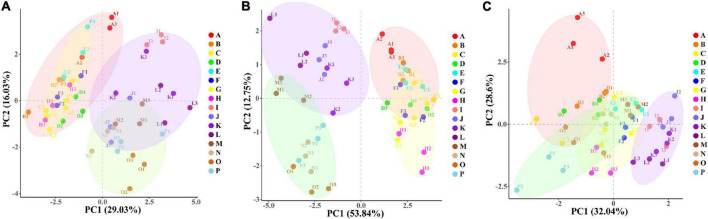
The PCA results of physiological indexes of *P. cathayana* seedlings inoculated with AM fungi under different P concentrations and drought stress. **(A)** PCA analysis of leaf; **(B)** PCA analysis of root; **(C)** PCA analysis of soil. The capital letters and different color markers on the right side of each figure represent A: NM + WW + P0, B: NM + WW + P50, C: NM + WW + P100, D: NM + WW + P150, E: AM + WW + P0, F: AM + WW + P50, G: AM + WW + P100, H: AM + WW + P150, I: NM + WD + P0, J: NM + WD + P50, K: NM + WD + P100, L: NM + WD + P150, M: AM + WD + P0, N: AM + WD + P50, O: AM + WD + P100; P: AM + WD + P150. The different colors ovals in each figure represent pink: NM + WW, yellow: AM + WW, purple: NM + WD, and green: AM + WD.

PC1 explained 29.03% and PC2 explained 16.03% of the total variables in the leaf (Figure 7A), and PC1 and PC2 explained 53.84 and 12.75% of the total variables in the root (Figure 7B). PCA analysis explained 45.06 and 66.59% of the physiological indexes in the leaf and root, respectively. The WW group (ABCDEFGH) and the WD group (IJKLMNOP) were relatively separated in the dimension of PC1; and each treatment of “NM + WW,” “AM + WW,” “NM + WD,” and “AM + WD” were distinguished according to different P levels, especially P0 and the other three P levels in the dimension of PC2. However, the NM and AM groups of the leaf were partially separated under WD conditions and completely overlapped under WW conditions. NM and AM groups of the root were partially separated under WW conditions and completely separated under WD conditions. PC1 and PC2, respectively, explained 32.04 and 28.6% of the total variables in the soil (Figure 7C). “NM + WD” was relatively separated from “NM + WW,” “AM + WW,” and “AM + WD” in the dimension of PC1, while “NM + WW + P0” was highly differentiated from other groups in the dimension of PC2.

**FIGURE 8:**
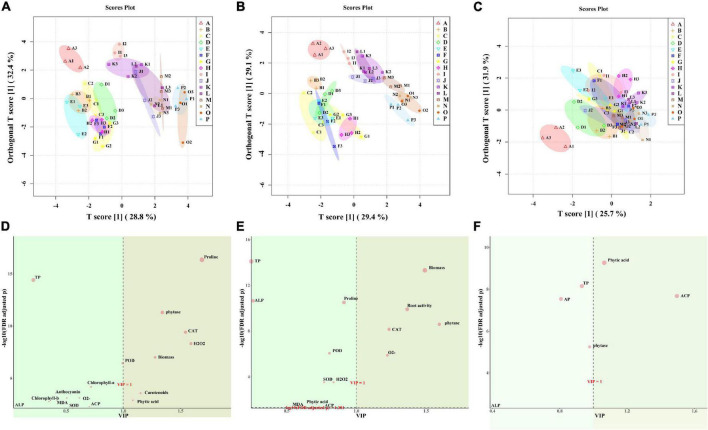
The OPLS-DA results of physiological indexes of *P. cathayana* seedlings inoculated with AM fungi under different P concentrations and drought stress. **(A,D)** OPLS-DA analysis of leaf; **(B,E)** OPLS-DA analysis of root; **(C,F)** OPLS-DA analysis of soil. The capital letters and different color markers on the right side of each figure represent A: NM + WW + P0, B: NM + WW + P50, C: NM + WW + P100, D: NM + WW + P150, E: AM + WW + P0, F: AM + WW + P50, G: AM + WW + P100, H: AM + WW + P150, I: NM + WD + P0, J: NM + WD + P50, K: NM + WD + P100, L: NM + WD + P150, M: AM + WD + P0, N: AM + WD + P50, O: AM + WD + P100; P: AM + WD + P150. The different colors ovals in each figure represent pink: NM + WW, yellow: AM + WW, purple: NM + WD, and green: AM + WD.

According to the OPLS-DA, the WW group (ABCDEFGH) and the WD group (IJKLMNOP) both in leaf and root could be well distinguished, and “NM + WW + P0” was clearly separated from the other 7 groups in the WW group (Figures 8A,B). While the 16 groups of the soil almost all overlapped, only “NM + WW + P0” was clearly separated from the other 15 groups (Figure 8C). The main substances that produced differences were proline, phytase, CAT, H_2_O_2_, biomass, carotenoids, and phytic acid in leaves and biomass, root activity, phytase, CAT, and O_2_^–^ in the roots. Then phytic acid and ACP were the main substances that produced a difference in the soil (VIP > 1, FDR < 0.05) (Figures 8D–F).

### Relationships between physiological indicators and the 14-3-3 gene expression

**FIGURE 9:**
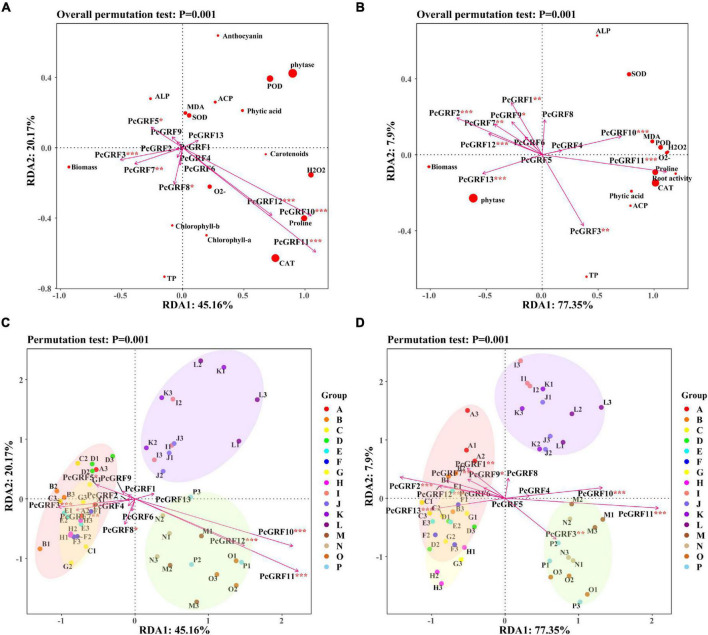
The RDA results of 14-3-3 gene expression and physiological indexes of *P. cathayana* seedlings inoculated with AM fungi under different P concentrations and drought stress. **(A,C)** RDA analysis of leaf; **(B,D)** RDA analysis of root. *P < 0.05, **P < 0.01, and ***P < 0.001. The capital letters and different color markers on the right side of each figure represent A: NM + WW + P0, B: NM + WW + P50, C: NM + WW + P100, D: NM + WW + P150, E: AM + WW + P0, F: AM + WW + P50, G: AM + WW + P100, H: AM + WW + P150, I: NM + WD + P0, J: NM + WD + P50, K: NM + WD + P100, L: NM + WD + P150, M: AM + WD + P0, N: AM + WD + P50, O: AM + WD + P100; P: AM + WD + P150. The different colors ovals in each figure represent pink: NM + WW, yellow: AM + WW, purple: NM + WD, and green: AM + WD.

Correlation analysis of the 14-3-3 gene and physiological indicators showed that *PcGRF10* and *PcGRF11* had a positive correlation with the physiological indicators both in the leaf and root (Supplementary Figure 9). The RDA results of the 14-3-3 gene expression and physiological indicators are shown in Figure 9. RDA1 and RDA2 explained 45.16 and 20.17% of the total variables, respectively. These explained 65.33% of the relationship between 14-3-3 gene expression and physiological indicators in *P. cathayana* leaves (Figure 9A). RDA1 and RDA2 explained 77.35 and 7.90% of the total variables, respectively, which explained 85.25% of the relationship between 14-3-3 gene expression and physiological indicators in *P. cathayana* roots (Figure 9B). *PcGRF3*, *PcGRF5*, *PcGRF7*, *PcGRF8*, *PcGRF10*, *PcGRF11*, and *PcGRF12* (*P* < 0.05) were the main genes that affected the physiological metabolism of leaves. *PcGRF1*, *PcGRF2*, *PcGRF3*, *PcGRF7*, *PcGRF9*, *PcGRF10*, *PcGRF11*, *PcGRF12*, and *PcGRF13* (*P* < 0.05) were the main genes that affected the physiological metabolism of roots. *PcGRF10*, *PcGRF11*, and *PcGRF12* in leaves and *PcGRF3*, *PcGRF10*, and *PcGRF11* in roots pointed to the “AM + WD” group, which may be the genes induced by AM fungi to be expressed in leaves and roots under drought stress, respectively (Figures 9C,D).

## Discussion

### Effects of P addition, drought, and arbuscular mycorrhizal fungi on P metabolism

The P content of leaves and roots increased with the increase of the P addition level in the NM + WW treatment; however, no significant change was observed at different P levels in the NM + WD treatment. This indicates that drought stress can lead to an imbalance in the absorption of mineral nutrients in plants except for the obvious effects on plant growth and development (Epie and Maral, 2018). Under drought conditions, increasing nutrient supply does not improve plant growth if sufficient amounts of nutrients are already present in the soil (Hu and Schmidhalter, 2005). Drought stress reduces the transfer of P from soil to roots and subsequently to stems by reducing the transpiration rate and altering the function of membrane transporters (Cramer et al., 2009). But AM fungi inoculation can enhance all these parameters (Xie et al., 2022). Our study found that the P content in leaves and roots of *P. cathayana* seedlings inoculated with AM fungi increased significantly with the P additional level under the WW and WD conditions, which was significantly higher than the no-inoculated seedlings. However, root biomass was not significantly different in the AM + WD treatment. Although longer roots mean more efficient use of P (Mori et al., 2016), it may not reflect the highest P uptake. Most of the P in plants colonized by AM fungi was derived from hyphae (Smith et al., 2004). The mycorrhizal pathway can contribute to more than 80% of P in plants under certain conditions as an important part of the P uptake system (Smith et al., 2003). Li et al. (2006) also found that more than 50% of P uptake by plants came from AM fungi even when soluble P fertilizers were applied. This suggests the existence of a functional AM pathway for P transfer to plants (Sato et al., 2019).

The total P content in soil is relatively rich, but a considerable part of soil P (50–80%) exists in an ineffective form and cannot be absorbed and utilized by plants (Starnes et al., 2008). Among them, phytic acid is one of the main organic P in soil, accounting for more than 50% of total soil P (Osborne and Rengel, 2002). P addition ameliorated the dry matter accumulation and caused the dilution effect of phytic acid concentration, which resulted in the complex relationship between phytic acid content and P supply in plants. Under adverse conditions such as drought, the increase in phytic acid concentration may be at least partially caused by a decrease in biomass (Thavarajah et al., 2010). Plants and root-associated microorganisms have evolved a series of strategies to enhance P absorption, including the release of organic acids (Zhang et al., 2016) and phosphatases (Robinson et al., 2012). Organic P is a potential pool as it can be hydrolyzed by phosphatase or phytase released by plants or microorganisms to obtain inorganic P (Kitayama, 2013; Zhang et al., 2014). In the “NM + WW” treatment, we found that the ACP and ALP of the root and rhizosphere soil at the P0 level were higher than the other three P levels, which indicated that low P stress would promote the phosphatase secretion of roots to activate the soil organic P. If available P is applied to the soil, the roots and soil microorganisms seem to produce less extracellular phosphatase and directly absorb soil inorganic P (Marklein and Houlton, 2012; Zhang et al., 2015). In the “NM + WD” treatment, the ALP activity of leaves and roots at the P0 level was significantly higher than other P levels, but there was no significant difference with other P levels in the rhizosphere soil. It indicates that soil water content plays an important role in root secretion of phosphatase and can further affect the mobilization of P in rhizosphere soil, thus affecting the P absorption by plants (Lambers et al., 2006). AM fungi can recruit phosphatase-producing microorganisms in the soil and promote their release, which could mineralize organic phosphate to promote plant P absorption (Zhang et al., 2018b). The results of previous studies showed that the contribution of AM fungi to P absorption was 49 and 77% under high P conditions (50 mg/kg) and low P conditions (24 mg/kg), respectively (Thingstrup et al., 2000). It was found that the activities of ALP and phytase in the rhizosphere soil inoculated with AM fungi under drought stress were higher than those of non-inoculated soil, and the ALP at P0, P50, and P150 levels and phytase at P0 and P150 were significantly higher than those of the non-inoculated soil. These indicated that the mycorrhizal seedlings of *P. cathayana* under drought stress mainly utilized ALP and phytase in the rhizosphere to hydrolyze organic P, to increase the absorption of P (Steffens et al., 2010). Zhang et al. (2018b) found that the bacteria recruited by AM fungi may produce ALP on the hyphal surface. Under the WD condition, AM fungi inoculation significantly decreased ALP activity in leaves and roots, but significantly increased in the rhizosphere soil. Previous research has shown that the microbial phosphatases may be easier to combine with phosphate compounds and release orthophosphate from soil organic P than plant phosphatases under drought stress (Tarafdar et al., 2001).

### Effects of P addition and arbuscular mycorrhizal fungi inoculation on reactive oxygen species homeostasis

The decrease of soil water potential hindered the water absorption of roots and ultimately reduced the growth of plants (Rubin et al., 2017). Although plants have an inherent resistance to drought stress, the adverse effects of drought can be minimized by an adequate and balanced supply of mineral nutrients. In the “NM + WD” treatment, the plant height and root activity at P50 and P100 levels were significantly higher than those at P0 and P150 levels. This indicates that P application can promote root growth, nutrient absorption, and water use efficiency to improve yield and alleviate the adverse effects of drought (Waraich et al., 2011). Similarly, the biomass of *P. cathayana* showed a trend of “first increase and then decrease” with the increase in P level. It only slightly increased at P50 and P100 levels and did not reach a significant level. This may be related to the abscission of the bottom leaves of taller plants after drought stress treatment. Combined with the results of plant height, root activity, and biomass, we found that P150 had a negative effect on the growth of *P. cathayana*, which may be due to the P toxicity caused by high levels of P (Rotaru and Sinclair, 2009). The positive effect of P on plant growth was attributed to ameliorated water relations and drought tolerance under drought conditions (Jin et al., 2006).

Previous studies have shown that fertilization can alleviate the adverse effects of drought on plant growth by promoting the regulation of water use efficiency and enhancing the activity of antioxidant enzymes (Khammari et al., 2012). In the “NM + WD” condition, the CAT enzyme activity and proline content of *P. cathayana* increased with the increase of the P level, especially in the roots. This indicates that drought showed an increase in the levels of osmotic substances and ROS scavengers compared with the combined stress of drought and P, which was attributed to the positive effect of P fertilizer on oxidative stress in the combined stress treatment (Meena et al., 2021). Similar results were observed in *Alnus cremastogyne* seedlings, suggesting that P application ameliorated the adverse effects of drought by enhancing antioxidant enzyme activity (Tariq et al., 2018). Carotenoids and anthocyanins were also part of the antioxidant defense system of plants, and their increase makes plants more resistant to oxidative stress (Mittler, 2002).

Previous reports have shown that early P supplementation can enhance the colonization of *Funneliformis mosseae* in *Catharanthus roseus*. Recently, Jiménez-Moreno et al. (2018) also demonstrated that P availability improves AM fungi colonization in olives. In this research, we found that the arbuscular colonization rate of *P. cathayana* seedlings showed a trend of “first increase and then decrease” with the increase of P concentration. The arbuscular colonization rate of P50 was significantly higher than other P levels under the WW condition, but there was no significant difference under the WD condition, and the arbuscular and hypha colonization in WD treatment was significantly higher than that in WW. The increase in nutrient concentration caused by the mycorrhizal effect is highly correlated with the level of mycorrhizal colonization (Sheng et al., 2013). The best treatment of P can improve the growth of AM fungi, and the best symbiotic relationship can transport a large amount of P to the host through external hyphae to promote plant growth (Dobo et al., 2016). AM fungi could regulate plant growth and development by surrounding roots with mycelium outside of free radicals (Gosling et al., 2006) and improving mineral nutrient uptake (Schnepf et al., 2011).

The improvement of the stress-tolerant mechanism of AM fungi symbiosis was usually related to the plant antioxidant activity (Baslam and Goicoechea, 2012). We found that POD and CAT activities in roots and CAT activities in leaves inoculated with AM fungi were significantly higher than those of non-inoculated plants under the WD condition. These results showed that the plants that were colonized with AM fungi showed higher enzyme activity under drought stress (Begum et al., 2019). However, they showed a decreasing trend with the increase of the P addition level. Our result supports previous findings that the addition of P to the soil usually reduces the development of AM fungi and then reduces the benefits of mycorrhizal symbiosis (Cavagnaro et al., 2005; Sheng et al., 2013). The excessive addition of soluble phosphate to soil reduced the mycorrhizal growth response of *P. cathayana* seedlings compared with the non-fertilized soil, which was not conducive to drought stress.

### Effects of 14-3-3s on P metabolism and reactive oxygen species homeostasis

The transcription and protein expression levels of the 14-3-3 gene in plants were affected by various abiotic stresses as reported in many previous studies (Liu et al., 2016; Xu et al., 2016). In this study, we found that the expression levels of *PcGRF2*, *PcGRF3*, and *PcGRF12* in leaves, and *PcGRF2* and *PcGRF7* in roots decreased with the increase of P addition levels in the “NM + WW” treatment, which may be the genes that respond to low P stress. In the “NM + WD” treatment, the expression levels of *PcGRF1*, *PcGRF2*, *PcGRF3*, *PcGRF4*, *PcGRF5*, *PcGRF6*, *PcGRF7*, *PcGRF8*, and *PcGRF9* in leaves, and *PcGRF1*, *PcGRF2*, *PcGRF3*, and *PcGRF5* in roots decreased with the increase of P addition levels, indicating that more 14-3-3’s expression was induced in plants under the dual stress of drought and low P (Huang et al., 2021). These results indicated that 14-3-3 proteins play a smart role in response to abiotic stress in plants (Xu et al., 2012a), but the expression of these genes did not have much effect on drought resistance. Our results showed that the relative expression levels of *PcGRF10*, *PcGRF11*, and *PcGRF12* in leaves, and *PcGRF10* and *PcGRF11* in roots were significantly higher under the WD condition than the WW, and their expression levels increased with the increase of P addition, which may help plants to absorb more P. The *PcGRF10* and *PcGRF11* expression levels of the “AM + WD” group in leaves were significantly higher than the “NM + WD” group, possibly suggesting that mycorrhizal induction could play a great role in plant 14-3-3 gene expression under the drought stress. Correlation analysis showed that *PcGRF10* and *PcGRF11* (ε group) in roots were significantly positively correlated with ACP, ALP, and phytic acid contents. Their expressions may relate to root production and secretion of phosphatase and the release of orthophosphates from soil organic P. Previous studies have found that the tomato 14-3-3 protein genes *TFT6* (non-ε group) and *TFT7* (ε group) are involved in the response to low P stress in the late and early stages, respectively. *TFT6* mainly promotes root growth by regulating leaf carbon distribution and increasing phloem sucrose transport and participates in the systematic response to P stress. *TFT7* acts directly in roots by activating root plasma membrane H^+^-ATPase to release more protons under P deficient conditions (Xu et al., 2012b). *Arabidopsis thaliana* overexpressing *GRF9* showed decreased starch accumulation compared with the wild type, suggesting that *GRF9* negatively regulates starch accumulation in the P-starvation metabolic pathway (Cao et al., 2007). Zhang et al. (2018a) also found that the heterologous *Arabidopsis GRF9* gene could confer resistance to P deficiency and promote fruit production in transgenic tomato plants. All these previous studies have demonstrated that different plant 14-3-3 subtypes have different functions under low P stress.

Lukaszewicz et al. (2002) showed that 14-3-3 protein affected the antioxidant activity of potato plants and that the overexpression of 14-3-3 protein was increased by 45% compared with the control plants. The antioxidant system of plants plays an important role in a variety of biotic and abiotic stresses. 14-3-3 protein interacted with ascorbate peroxidase (APX), a key enzyme that can remove H_2_O_2_ and prevent damage under oxidative stress or water shortage (Yan et al., 2004). Transgenic rice that silenced *GF14e* showed a high level of disease resistance compared with non-silenced plants, which was associated with higher basal expression of the defense response peroxidase gene (*POX22.3*) and accumulation of ROS (Manosalva et al., 2011). Tobacco (*Nicotiana tabacum*) overexpressed *Lr14-3-3* of *Lilium regale* Wilson showed up-regulation of the antioxidant enzymes activities, including SOD, glutathione S-transferase (GST), and APX (Li et al., 2014). Stress-related marker genes involved in the ABA signaling pathway, ROS scavenging system, and ion transporter were up-regulated in tobacco overexpressing 14-3-3 protein gene *BdGF14d* from *Brachypodium distachyon* under saline-alkali stress, which enhanced the CAT and POD activities of tobacco (He et al., 2017). In this study, the correlation analysis showed that *PcGRF10* and *PcGRF11* had a very significant positive correlation with CAT, proline, and carotenoids in leaves and with SOD, POD, CAT, proline, and root activity in roots (*P* < 0.001). As antioxidant defense and osmotic regulation substances, they played an important role in the ROS homeostasis of plants. ROS such as O_2_^–^ and H_2_O_2_ in plants can be removed by enzymatic antioxidants (SOD, POD, and CAT) (Hanin et al., 2016) or non-enzymatic antioxidants (carotenoids) (AbdElgawad et al., 2016). Cell expansion, osmotic potential, and membrane stability could be maintained through osmotic regulatory substances accumulated in the cytosol (Keunen et al., 2013). These results suggest that *PcGRF10* and *PcGRF11* may be involved in the regulation of antioxidant enzyme activities and osmotic substances during drought resistance (Han et al., 2022).

A single 14-3-3 protein gene can act as the regulator of several different abiotic stresses and may mediate crosstalk in several disparate signaling pathways. At present, the 14-3-3 protein gene was found to be induced by AM fungi under drought stress, which is regulated by the plant ABA signaling pathway (Xu et al., 2018) and can cross-link with d-inositol 3-phosphate synthase (IPS) (Li et al., 2016). Studies on the role of 14-3-3 genes in P metabolism and drought stress of plants inoculated with AM fungi are scarce. It is well known that individual genes, especially transcription factors, may play a role in several different signaling pathways, which can be inferred from their expression profiles induced by various stress factors (Chen et al., 2012). As mentioned above, we found that *PcGRF10* and *PcGRF11* have a positive correlation with the antioxidant system, osmotic regulation, and P metabolism, and they have a better correlation after inoculation with AM fungi.

## Conclusion

In summary, the P content in leaves and roots was significantly increased after inoculation with AM fungi, but the benefit of AM fungi on root P uptake under WD conditions is limited by the level of P addition. P addition reduced ALP activity in leaves and roots under drought stress. The roots of *P. cathayana* may produce and secrete more ALP and phytase to decompose organic P in the soil under drought and P stress, and the activities in the rhizosphere soil inoculated with AM fungi were stronger. AM fungi improved the adverse effects of *P. cathayana* on drought stress by up-regulating the antioxidant system (POD and CAT), osmotic accumulation (proline), and root activity. P did not play a significant role in ROS homeostasis. The 14-3-3 protein genes, *PcGRF10* and *PcGRF11*, have a positive correlation with the ROS homeostasis regulator (leaf: proline and CAT; root: POD, CAT, and proline), especially after inoculation with AM fungi. The current study contributes to the establishment of a mechanistic framework to improve the rational application of AM fungi and P fertilizers in poplar to cope with drought in arid and semi-arid regions.

## Data availability statement

The original contributions presented in this study are included in the article/Supplementary material, further inquiries can be directed to the corresponding authors.

## Author contributions

YH performed the experiments, analyzed the data, wrote the first manuscript, and modified it. WZ prepared the material and collected the samples. TX conceived the experiments, curated the data, revised the manuscript, and modified the language. MT acquired the funding, administrated the project, and supervised it. All authors contributed to the article and approved the submitted version.

## Footnotes

1
https://www.bioincloud.tech
