# Supplementary material for: Effect of arbuscular mycorrhizal fungi and phosphorus on drought-induced oxidative stress and 14-3-3 proteins gene expression of Populus cathayana
Source: Front Microbiol. 2022 Aug 11;13:934964. doi: 10.3389/fmicb.2022.934964 (PMC9403482; doi:10.3389/fmicb.2022.934964)
Supplement: Supplementary file 1 [file Data_Sheet_1.pdf]

Supplementary Material

Supplementary Table 1 Gene-specific primers used for qRT-PCR.

Gene	Forward Primers (5'-3')	Reverse Primers (5'-3')
PcGLL	CTCTCATTGAGCCGGCAAAT	CCCCCCTTCAAGCATAAGG
Pctublin	GATTTGTCCCTCGCGCTGT	TCGGTATAATGACCCTTGGCC
PcGRF1	ATTGGCTCTTAACTTCTCTGTCTTC	ACGTATCCAGCTCCGCAATG
PcGRF2	CCAACGGCTTCTTCTGGTGA	GAGACGGATTGGGTGAGTGG
PcGRF3	TTGCCAAGCAGGCTTTTGAC	AGTGTGCAACCTTGGGGAAA
PcGRF4	TCTCCAACTGAACCCATCACG	ATCCTCCATGAAGCCCTCCT
PcGRF5	GAAGCTGGGCTCAGCAAGAT	TGCTCTCAGCAGCTTCCTTC
PcGRF6	TCTGGCTGAGTTCAAGGCTG	CCTCAAATGCCTGTTTCGCC
PcGRF7	CTGATGTTTGTGCTAGTATTTTGAGGT	GCAATATCCTGAGCAGCCTTG
PcGRF8	ATACCCGGGGAATCCTCTGT	GCCCGTTCAGGAGAGTTCAT
PcGRF9	TTCGACTGGGTTTGGCTCTT	TCTGGTCTTCTCCATCCTCAG
PcGRF10	CCTGGCACTCAACTTTTCCG	CTGTAGAGTGCTCACCTCCTTC
PcGRF11	AACTCCCCTGAAAGGGCCTG	TCAGTTTTGCTTTACCTCTGCC
PcGRF12	CCGAGCGCTATGACGAGA	AGACATGATCCGCCAAGAAGC
PcGRF13	GCGCTATGATGAAATGGTTGAGAG	CTGGCAGTAACCCTTGATCAGT

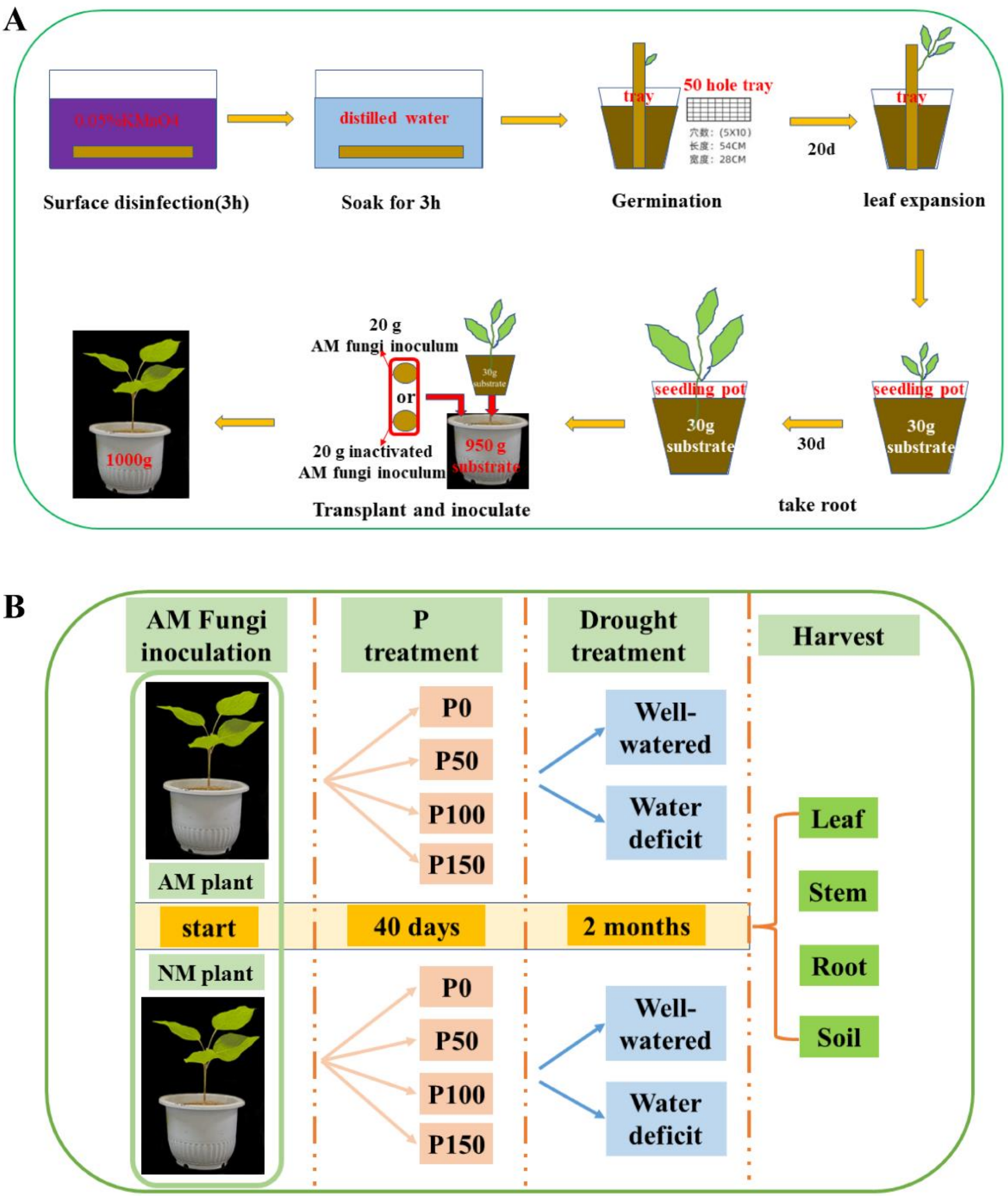

Supplementary Figure 1. Schematic diagram of seedling raising process **(A)** and experimental design **(B)**. P0, P50, P100, and P150 represent different P content of 0, 50, 100, and 150 mg, respectively.

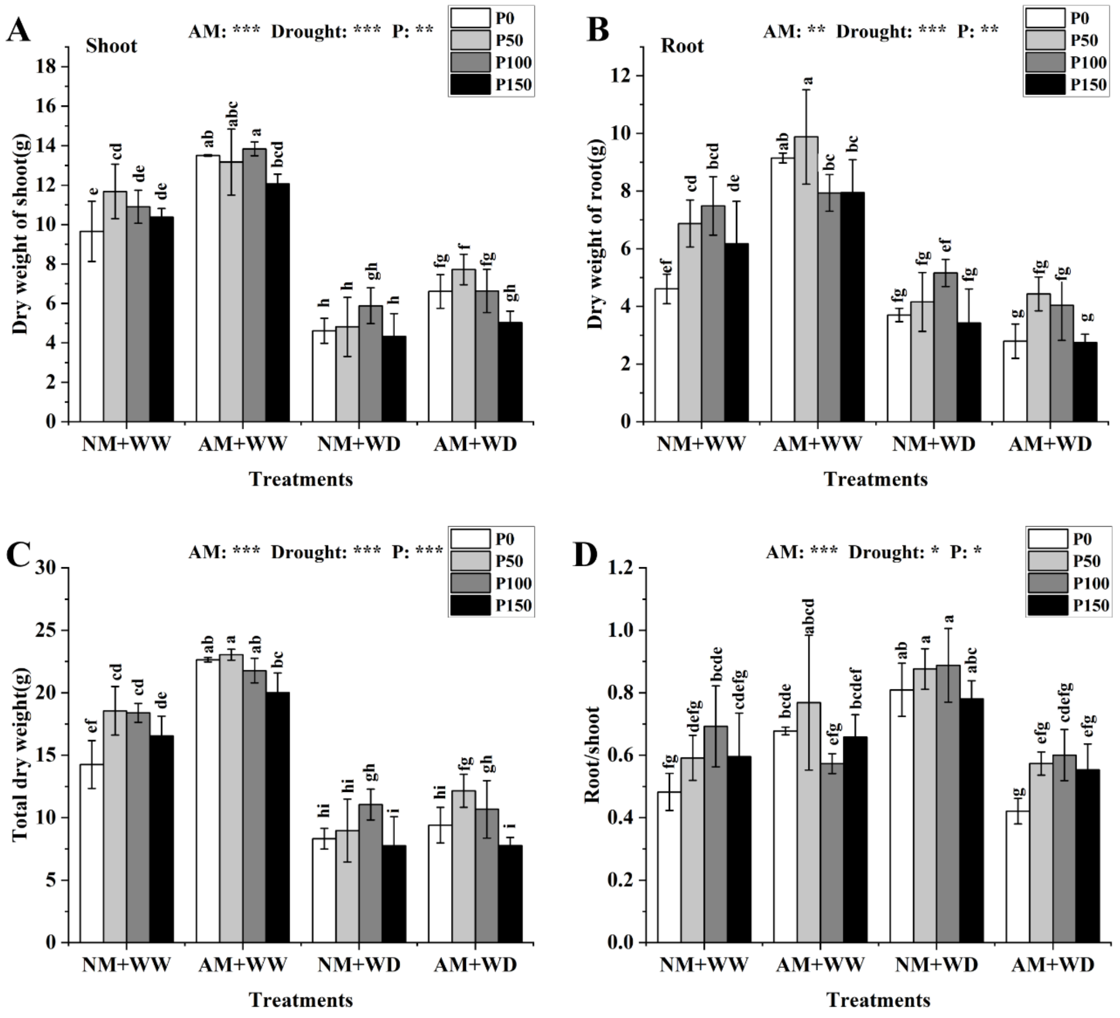

Supplementary Figure 2. Biomass of *P. cathayana* seedlings inoculated with AM fungi under different P addition and water treatment. **(A)** aboveground biomass; **(B)** underground biomass; **(C)** total biomass; **(D)** root-to-shoot biomass ratio.

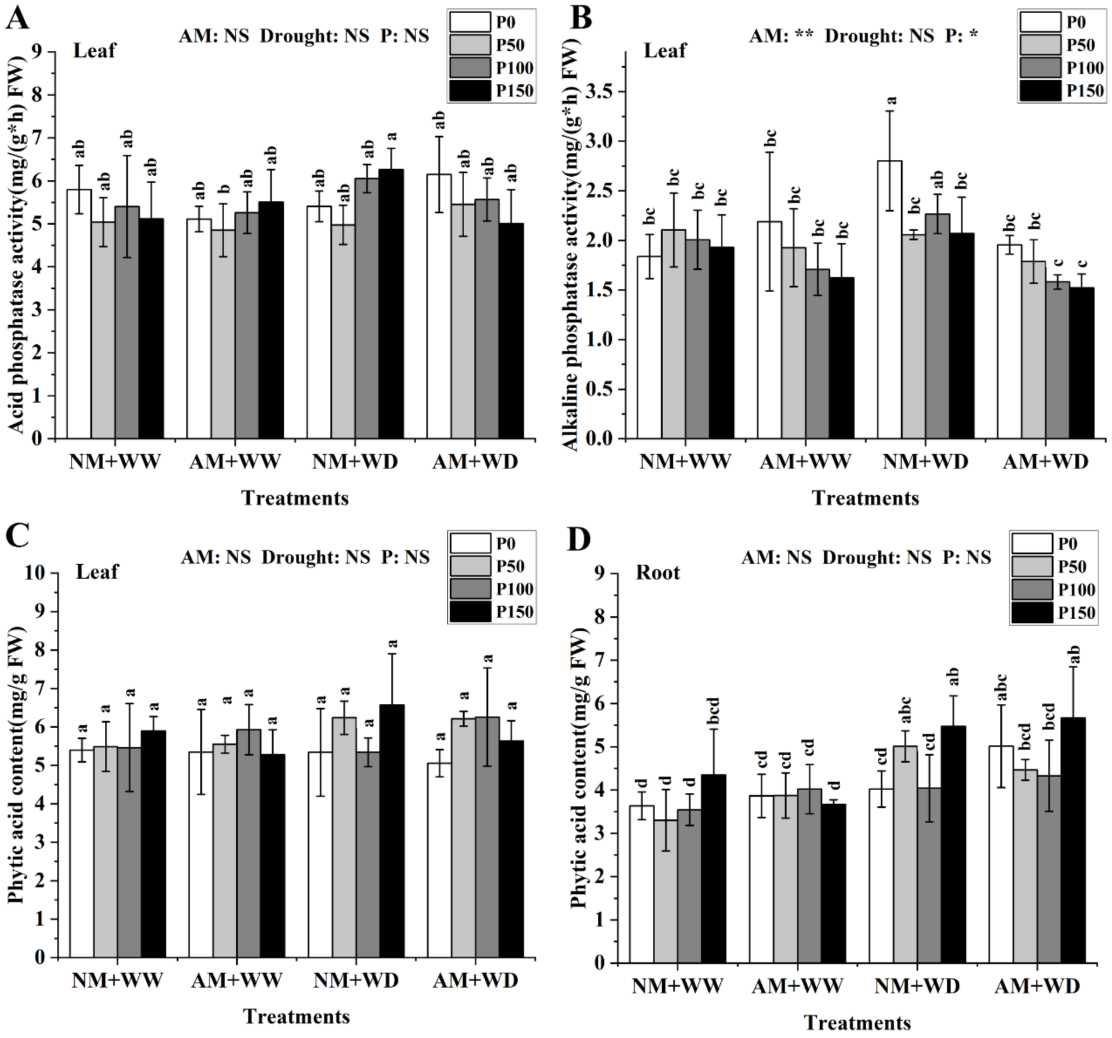

Supplementary Figure 3. Phosphatase content and phytic acid content of *P. cathayana* seedlings inoculated with AM fungi under different P addition and water treatment. **(A)** acid phosphatase activity in leaf; **(B)** alkaline phosphatase activity in leaf; **(C)** phytic acid content in leaf; **(D)** phytic acid content in root.

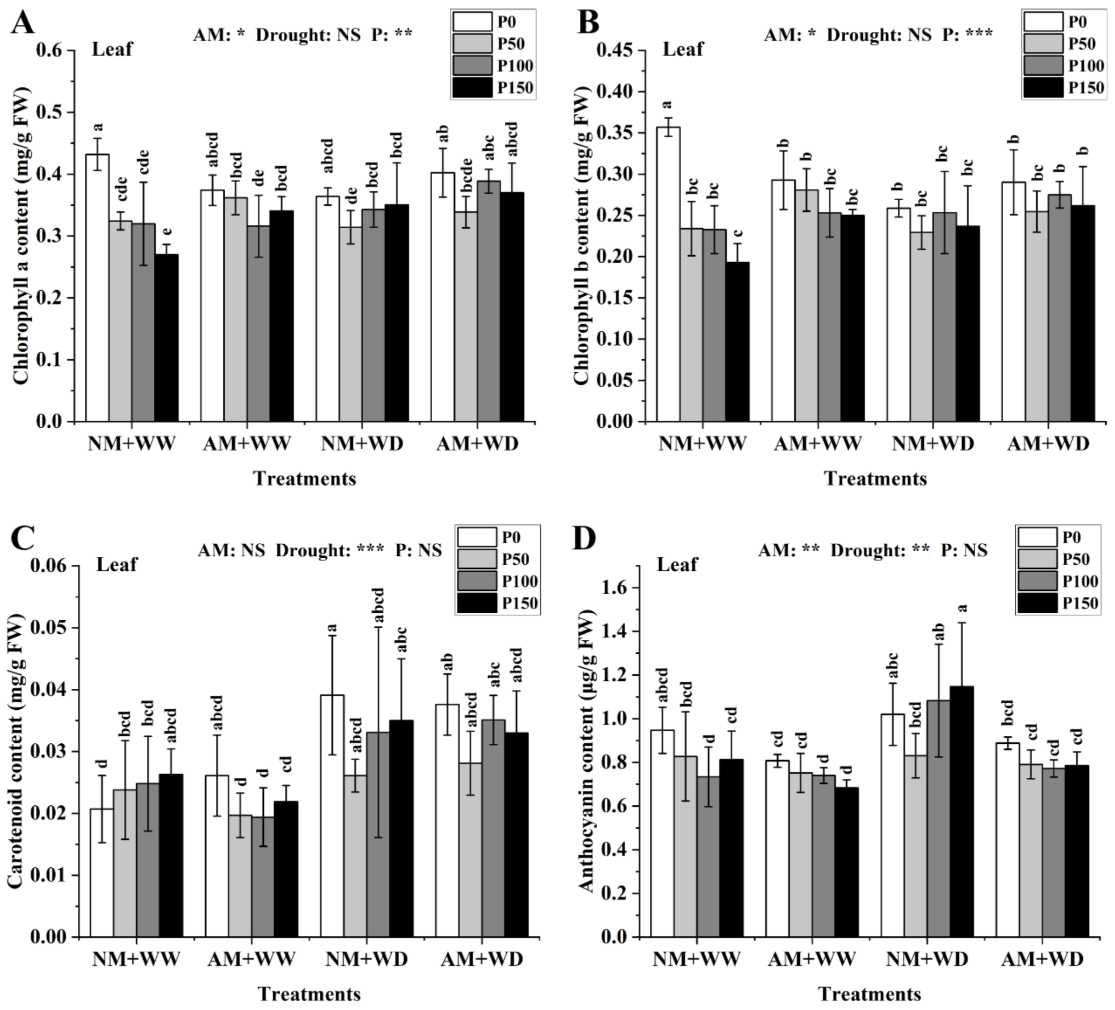

Supplementary Figure 4. Pigment content in the leaves of *P. cathayana* seedlings inoculated with AM fungi under different P addition and water treatment. **(A)** chlorophyll a content; **(B)** chlorophyll b content; **(C)** carotenoid content; **(D)** anthocyanin content.

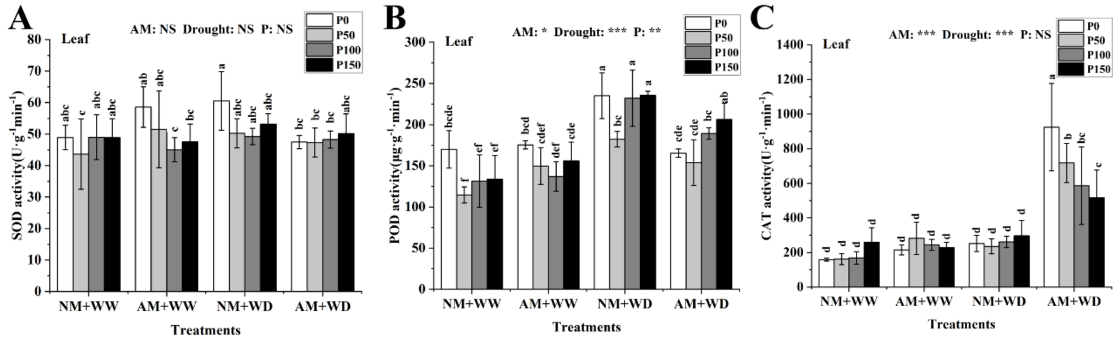

Supplementary Figure 5. Antioxidant enzyme activity in the leaves of *P. cathayana* seedlings inoculated with AM fungi under different P addition and water treatment. **(A)** SOD activity; **(B)** POD activity; **(C)** CAT activity.

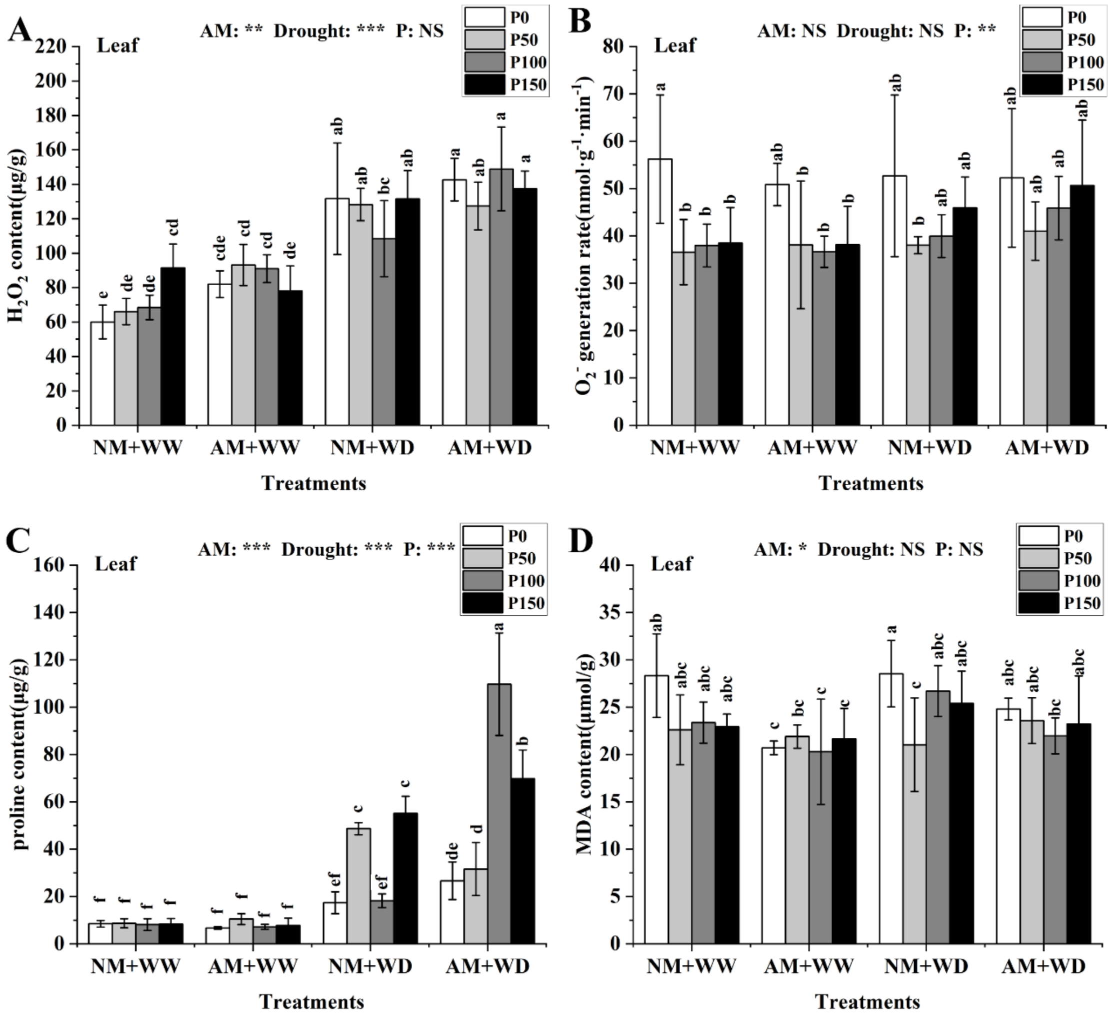

Supplementary Figure 6. Oxidative damage material in the leaves of *P. cathayana* seedlings inoculated with AM fungi under different P addition and water treatment. **(A)** H_2O_2 content; **(B)** O_2^- generation rate; **(C)** proline content; **(D)** MDA content.

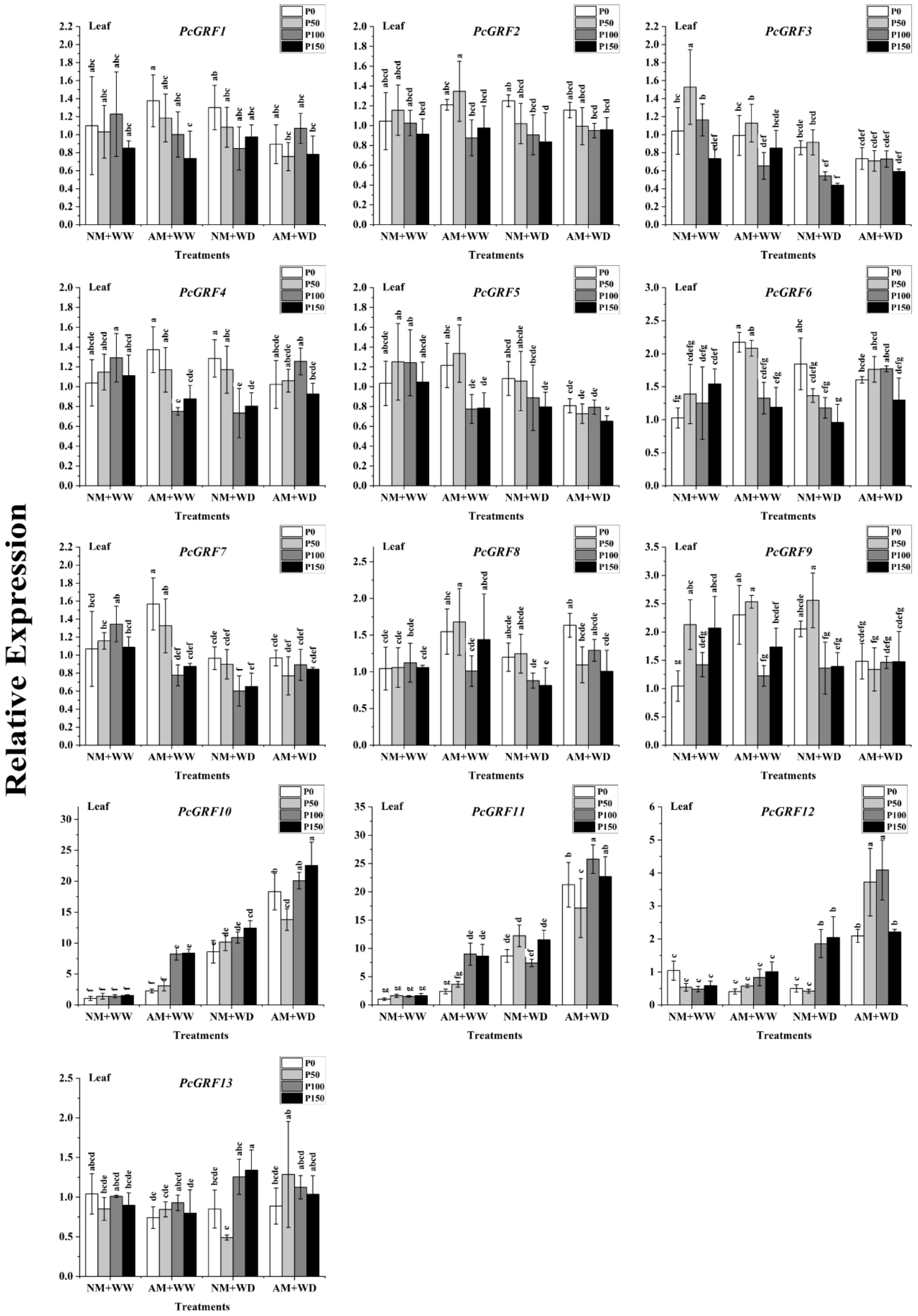

Supplementary Figure 7. Relative expressions of 14-3-3 protein genes in leaves of *P. cathayana* seedlings inoculated with AM fungi under different P addition and water treatment.

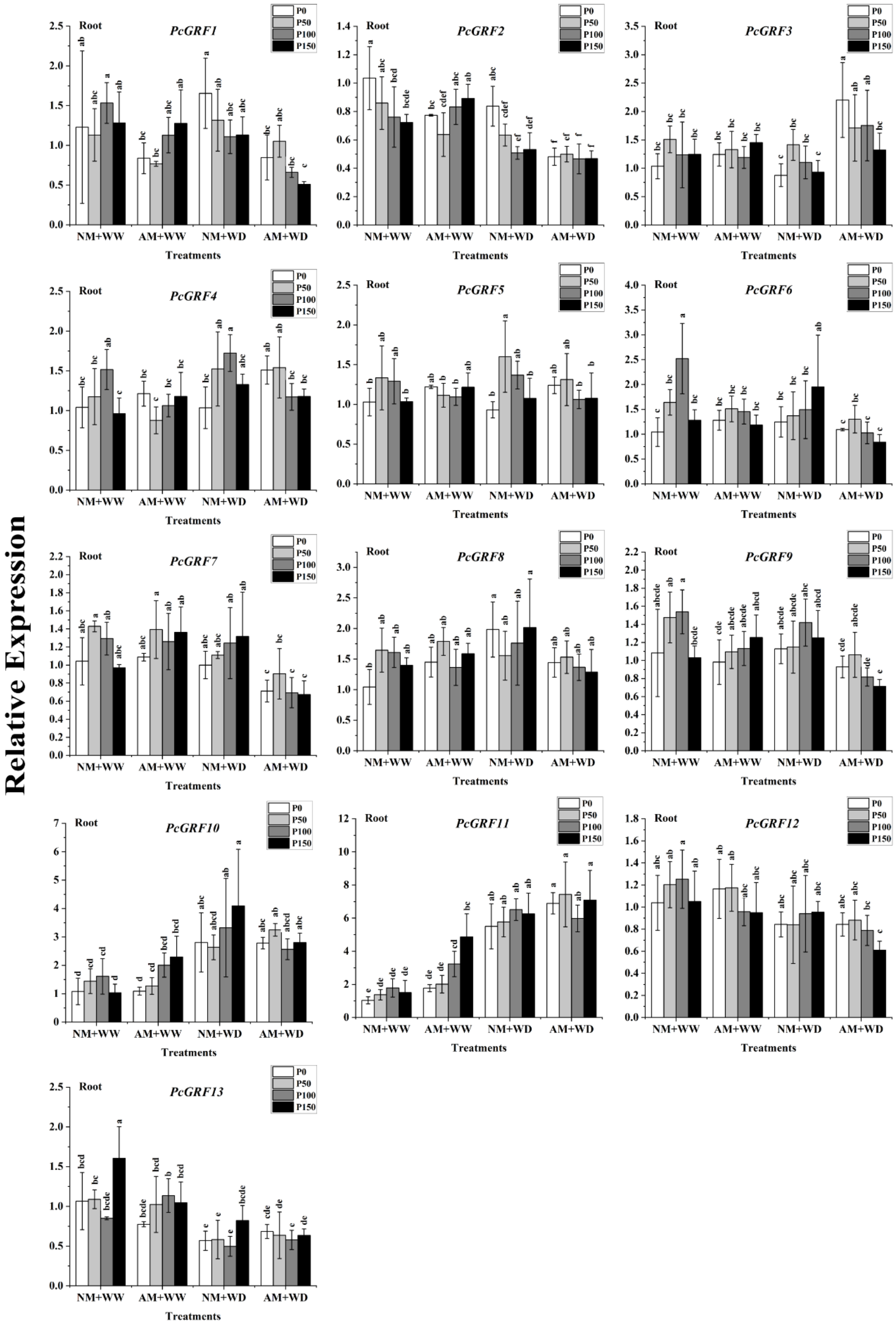

Supplementary Figure 8. Relative expressions of 14-3-3 protein genes in roots of *P. cathayana* seedlings inoculated with AM fungi under different P addition and water treatment.

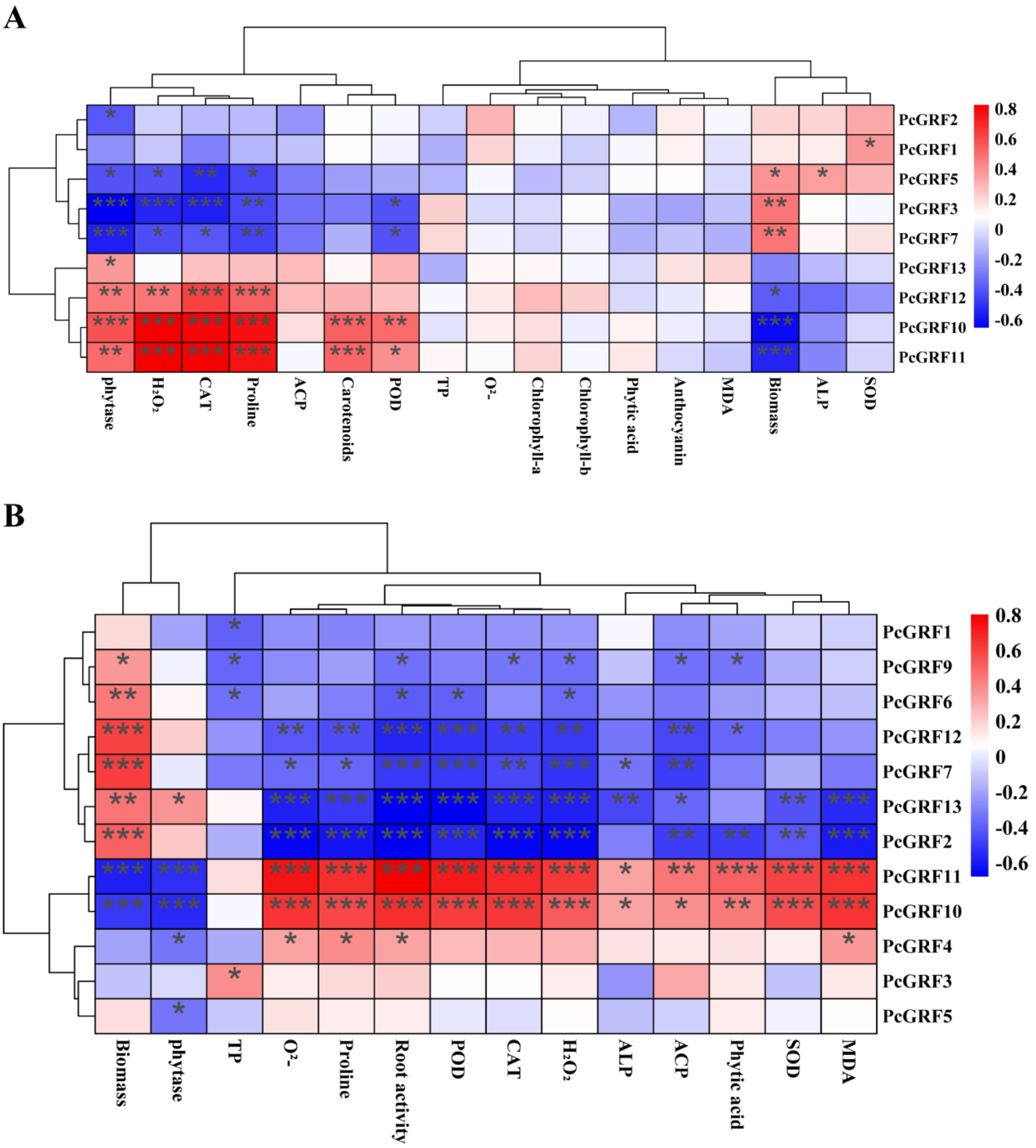

Supplementary Figure 9. (A) leaf and **(B)** root correlation heatmap of gene expression and physiological indicators of *P. cathayana* seedlings inoculated with AM fungi under different P addition and water treatment. $*P < 0.05$, $**P < 0.01$ and $***P < 0.001$.
